# Editorial for Special Issue—“Early-Stage Drug Discovery: Advances and Challenges”

**DOI:** 10.3390/ijms24076516

**Published:** 2023-03-30

**Authors:** Wolfgang Sippl

**Affiliations:** Institute of Pharmacy, Martin-Luther University of Halle-Wittenberg, Kurt-Mothes-Str. 3, 06122 Halle, Germany; wolfgang.sippl@pharmazie.uni-halle.de

The development of a new drug from the first hit to the launch of an approved product is a complex process that usually take around 12–15 years and costs more than USD 1–2 billion. The idea for a new target can come from a variety of sources, including clinical research, phenotypic studies, structural biology, and the use of in silico methods. It can take many years to validate a target sufficiently to initiate a costly drug discovery program. Once a target is validated, numerous processes must be carried out to identify molecules with suitable properties to be accepted as drug candidates for clinical trials. The application of in silico methods, such as structure-based design, molecular dynamics simulations, artificial intelligence, and machine learning, has become an essential part of the early drug discovery process. However, the development of novel biophysical and functional cellular assay methods could also help drive the selection of appropriate targets and lead structures.

In this Special Issue we focused on the early preclinical phase of drug discovery with all its different disciplines. The goal was to present new and interesting lead candidates, pharmacological insights and innovative methods. The current Special Issue is the result of a call for papers that was sent out in late 2021 and resulted in several submissions, from which ten papers (eight original research articles and two reviews) were published that covered novel developments in the design strategies of new lead compounds, structure–activity relationships, in vitro and in silico analyses, and pharmacokinetic properties. These articles not only summarize the recent developments in different perspectives of drug discovery but also present a wealth of information on novel lead compounds and an exploration of their biological effects against various targets of medicinal importance. Herein, the contributions to this Special Issue are summarized.

Günther et al. developed a novel ensemble stacked machine learning (ML) model to predict hyper-reactive druggable cysteines, named HyperCys, in protein structures [1]. Based on an intensive and careful inspection of available structural data, the authors devised a validated machine-learning-based prediction model. The developed model was found to be more accurate for predicting hyper-reactive druggable cysteines than previously described models. HyperCys might be an effective tool for discovering new potential reactive cysteines in a wide range of nucleophilic proteins and could provide an important contribution to the design of targeted covalent inhibitors.

Barinka et al. carried out a detailed comparison of the inhibitory activities of hydroxamic acids and difluoromethyl-1,3,4-oxadiazole against histone deacetylase subtypes [2]. Authors used enzymatic in vitro studies as well as a target engagement test in cell lines to analyze subtype selectivity. The reported results clearly show that the off-target effects of histone deacetylase 6 (HDAC6) inhibitors must be considered before attributing observed physiological readouts solely to HDAC6 inhibition. Moreover, given their unparalleled specificity, the authors propose the use of oxadiazole-based inhibitors as more appropriate research tools for investigating HDAC6 biology or as leads in the development of truly HDAC6-specific compounds in the treatment of human diseases. This study is relevant because the authors critically examine the most commonly used compounds in the literature—selective HDAC inhibitors for their actual cellular selectivity.

In the in silico study reported by Indiveri et al., the large amino acid transporter 1 (LAT1), which is overexpressed in several human cancers, was studied [3]. The authors generated outward–open and inward–occluded homology models of LAT1. The models were used to define the substrate and inhibitor/protein interaction during the transport cycle. The authors found that the binding scores for the substrate depend on the conformation, with the occluded states as the crucial steps affecting the substrate affinity. The validated models of LAT1 could be used to guide the identification of potential inhibitors through in silico screening.

Nicolaes et al. describe the development of novel inhibitors against vascular calcification (VC). VC is an important contributor and prognostic factor in the pathogenesis of cardiovascular diseases [4]. The authors concentrated on the neutral sphingomyelinase 2 (nSMase2 or SMPD3), which plays a key role in VC. In search for novel druglike small molecule inhibitors of nSMase2, we used a virtual ligand screening to identify potential ligands. Based on an in silico collection of 486,844 small druglike molecules, the authors carried out a stepwise structure-based virtual screening and identified 52 individual hits. Further analysis showed that five compounds had an IC_50_s lower than 2 µM and were able to decrease human primary extracellular vesicles by vascular smooth muscle cells release and calcification in vitro. The identified molecules represent new classes of nSMase2 inhibitors that can be used for further optimization studies to explore therapeutic or prophylactic treatment with VC.

In the study by Chong et al., a machine-learning-based model was developed to classify mitochondrially toxic and non-toxic compounds [5]. The initiation and development of chemical-induced organ damage are associated with mitochondrial dysfunction, among several adverse effects, and some drugs, e.g., troglitazone, have been withdrawn from the market because of mitochondrial toxicity. For their model development, the authors considered different molecular descriptors and machine learning methods. The selected features used with the CatBoost learning algorithm achieved a prediction accuracy of 85% in a 10-fold cross-validation and 87% in independent testing. The proposed model illustrated an improved prediction accuracy when compared with the existing state-of-the-art method available in the literature.

In the study by Hanke et al., a kinase subfamily, thus far understudied, was analyzed and novel inhibitors were developed [6]. The studied PCTAIRE (kinase subfamily with sequence motif in the kinase αC helix that is conserved in CDK17 and CDK18) subfamily belongs to the CDK (cyclin-dependent kinase) family, which exhibits a highly conserved binding pocket. The authors focused on the subtype CDK16, which is disregulated in several diseases, including breast, prostate, and cervical cancer. The N-(1H-pyrazol-3-yl)pyrimidin-4-amine moiety, discovered from a promiscuous inhibitor, was used to develop selective compounds for PCTAIRE. The cellular potency was determined for all new synthesized compounds using NanoBRET technology. Chemical optimization steps led to a promising compound (43d), which exhibited a nanomolar cellular potency for CDK16 (EC_50_ = 33 nM) and the other members of the PCTAIRE. A biophysical screen against a representative panel of approximately 100 kinases exhibited a selective inhibition over the other kinases. In a viability assessment, the best compound decreased the cell count in a dose-dependent manner and revealed a G2/M phase cell cycle arrest caused by the inhibition of CDK16. This study nicely showed how structure-based methods, modern medicinal chemistry, and informative cellular assay methods can be used to obtain valid chemical probes for understudied kinases.

Chen et al. report on the development of novel compounds to block the entry of SARS-CoV-2 into host cells [7]. The authors identified a collection of aloperine derivatives that inhibited the entry of SARS-CoV-2 (D614G variant of spike protein) virus with IC_50_s in the submicromolar range. The best compound was also active against several other SARS-CoV-2 variants, including Delta and Omicron. The results of a confocal microscopy study suggest that the compound inhibited viral entry before fusion to the cell or endosomal membrane. In summary, the authors showed that aloperine is a privileged scaffold that can be used to develop novel anti-SARS-CoV-2 entry inhibitors.

In a study by Sippl et al., the structure-based development of novel class I histone deacetylase inhibitors (HDAC) for the treatment of leukemic diseases is reported [8]. Class I histone deacetylases (HDACs) are key regulators of cell proliferation and they are frequently dysregulated in cancer cells. The novel HDAC inhibitors were synthesized using a 2-aminobenzamide moiety as a zinc-binding group connected with a central (piperazin-1-yl)pyrazine or (piperazin-1-yl)pyrimidine moiety. The detailed enzymatic in vitro testing against HDAC subtypes showed that the most promising compounds were found to be highly selective against HDAC1, 2, and 3. Molecular docking studies and MD simulations were performed to rationalize the in vitro data and to deduce a complete structure activity relationship (SAR) analysis. Cellular studies of the best compounds showed HDAC1 target engagement, high potency against human acute myeloid leukemia (AML) and erythroleukemic cancer (HEL) cells, and low toxicity against human embryonic HEK293 cells. The best compounds were found to be superior to the clinically tested class-I HDAC inhibitor entinostat. The developed specific HDACi can be used to further study the potential to eliminate blood cancer cells of various origins.

The two included review articles address two hot topics in current drug discovery research, artificial intelligence (AI) methods as well as proteolysis targeting chimera (PROTACs). The review by Sony et al. is an excellent compilation of AI methods currently used in drug design [9]. It also discusses the upcoming use of AI to refine and strengthen drug discovery. The aim of the second review, submitted by Vicente and Salvador, is to analyze and discuss the characteristics of MDM2 (mouse double minute 2 homolog)-based PROTACs developed for the degradation of oncogenic proteins and to understand the potential that they have as future anticancer drugs [10].

In summary, the current Special Issue encompasses both experimental and in silico research. Moreover, in silico approaches were successfully exploited not only for the structure-based design of potential inhibitors but also for the elucidation of target binding, cellular potency and the search for reactive cysteines in proteins. Overall, the research presented in this Special Issue effectively demonstrates how different experimental and in silico methods are able to contribute to the development of lead structures and chemical probes for targets that have rarely been explored.

## References

[1] Gao M., Günther S. (2023). HyperCys: A Structure- and Sequence-Based Predictor of Hyper-Reactive Druggable Cysteines. Int. J. Mol. Sci..

[2] Ptacek J., Snajdr I., Schimer J., Kutil Z., Mikesova J., Baranova P., Havlinova B., Tueckmantel W., Majer P., Kozikowski A. (2023). Selectivity of Hydroxamate- and Difluoromethyloxadiazole-Based Inhibitors of Histone Deacetylase 6 In Vitro and in Cells. Int. J. Mol. Sci..

[3] Brunocilla C., Console L., Rovella F., Indiveri C. (2023). Insights into the Transport Cycle of LAT1 and Interaction with the Inhibitor JPH203. Int. J. Mol. Sci..

[4] Pavlic A., Poelman H., Wasilewski G., Wichapong K., Lux P., Maassen C., Lutgens E., Schurgers L.J., Reutelingsperger C.P., Nicolaes G.A.F. (2023). Inhibition of Neutral Sphingomyelinase 2 by Novel Small Molecule Inhibitors Results in Decreased Release of Extracellular Vesicles by Vascular Smooth Muscle Cells and Attenuated Calcification. Int. J. Mol. Sci..

[5] Jaganathan K., Rehman M.U., Tayara H., Chong K.T. (2022). XML-CIMT: Explainable Machine Learning (XML) Model for Predicting Chemical-Induced Mitochondrial Toxicity. Int. J. Mol. Sci..

[6] Amrhein J.A., Berger L.M., Tjaden A., Krämer A., Elson L., Tolvanen T., Martinez-Molina D., Kaiser A., Schubert-Zsilavecz M., Müller S. (2022). Discovery of 3-Amino-1H-pyrazole-Based Kinase Inhibitors to Illuminate the Understudied PCTAIRE Family. Int. J. Mol. Sci..

[7] Huang L., Zhu L., Xie H., Goodwin J.S., Rana T., Xie L., Chen C.H. (2022). Quinolizidines as Novel SARS-CoV-2 Entry Inhibitors. Int. J. Mol. Sci..

[8] Ibrahim H.S., Abdelsalam M., Zeyn Y., Zessin M., Mustafa A.M., Fischer M.A., Zeyen P., Sun P., Bülbül E.F., Vecchio A. (2022). Synthesis, Molecular Docking and Biological Characterization of Pyrazine Linked 2-Aminobenzamides as New Class I Selective Histone Deacetylase (HDAC) Inhibitors with Anti-Leukemic Activity. Int. J. Mol. Sci..

[9] Sarkar C., Das B., Rawat V.S., Wahlang J.B., Nongpiur A., Tiewsoh I., Lyngdoh N.M., Das D., Bidarolli M., Sony H.T. (2023). Artificial Intelligence and Machine Learning Technology Driven Modern Drug Discovery and Development. Int. J. Mol. Sci..

[10] Vicente A.T.S., Salvador J.A.R. (2022). MDM2-Based Proteolysis-Targeting Chimeras (PROTACs): An Innovative Drug Strategy for Cancer Treatment. Int. J. Mol. Sci..

